# The genome sequence of the seven-spotted ladybird, *Coccinella septempunctata *Linnaeus, 1758

**DOI:** 10.12688/wellcomeopenres.17346.1

**Published:** 2021-11-24

**Authors:** Liam Crowley, University of Oxford and Wytham Woods Genome Acquisition Lab, Darwin Tree of Life Barcoding collective, Wellcome Sanger Institute Tree of Life programme, Wellcome Sanger Institute Scientific Operations: DNA Pipelines collective, Tree of Life Core Informatics collective, Darwin Tree of Life Consortium

**Affiliations:** 1 Department of Zoology, University of Oxford, Oxford, UK

**Keywords:** Coccinella septempunctata, seven-spotted ladybird, genome sequence, chromosomal

## Abstract

We present a genome assembly from an individual female *Coccinella septempunctata *(the seven-spotted ladybird; Arthropoda; Insecta; Coleoptera; Coccinellidae). The genome sequence is 399 megabases in span. The majority (99.96%) of the assembly is scaffolded into 9 chromosomal pseudomolecules, with the X sex chromosome assembled.

## Species taxonomy

Eukaryota; Metazoa; Ecdysozoa; Arthropoda; Hexapoda; Insecta; Pterygota; Neoptera; Endopterygota; Coleoptera; Polyphaga; Cucujiformia; Coccinellidae; Coccinellinae; Coccinellini; Coccinella; *Coccinella septempunctata* Linnaeus, 1758 (NCBI:txid41139).

## Background

The 7-spot ladybird, *Coccinella septempunctata* Linnaeus, 1758, is an iconic species of ladybird and one of the most common in the UK and across Europe. It is widespread and abundant throughout its native range of Europe, Asia and North Africa, although it’s distribution trend in the UK is decreasing ( Roy & Brown, 2018). It can be found across a wide range of habitats including gardens and agricultural land. Adults are large (5–8 mm), conspicuously marked species with vivid red elytra marked with 7 black spots. The head, pronotum and legs are black. The scarce 7-spot ladybird, *Coccinella magnifica*, is very similar, but can be distinguished by its larger black spots, and additional pair of white markings below the legs on the underside. The 7-spot ladybird is a predatory species, feeding on a wide range of aphid species both as a larva and as an adult. It overwinters as an adult in among foliage, dead plant material and leaf litter. The broad geographic success of this species may be underpinned by its ecological plasticity based on both genetic and phenotypic polymorphisms ( Hodek *et al.*, 2013). It has been repeatedly introduced to North America as a biological control agent against aphids in agricultural systems.

## Genome sequence report

**Figure 1.**
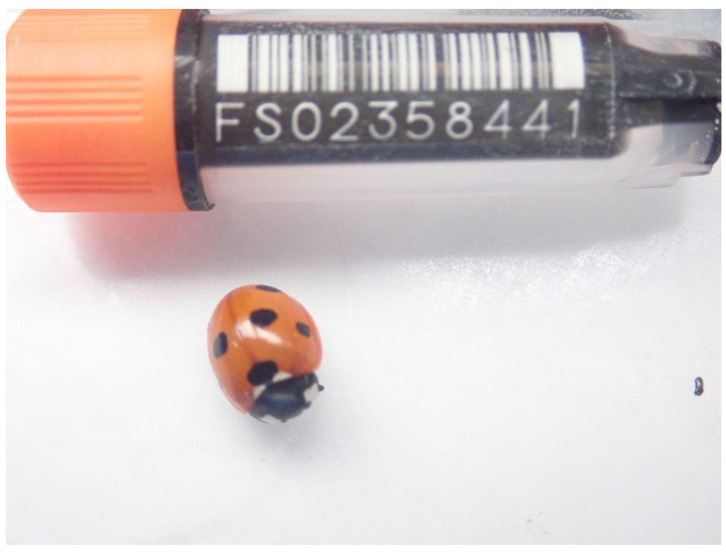
Image of the icCocSept1 specimen captured prior to preservation and processing.

The genome was sequenced from one female *C. septempunctata* ( Figure 1) collected from Wytham Farm, Oxfordshire (biological vice-county: Berkshire), UK (latitude 51.779, longitude -0.317). A total of 76-fold coverage in Pacific Biosciences single-molecule long reads and 89-fold coverage in 10X Genomics read clouds were generated. Primary assembly contigs were scaffolded with chromosome conformation Hi-C data. Manual assembly curation corrected 78 missing/misjoins and removed 10 haplotypic duplications, reducing the assembly length by 2.60% and the scaffold number by 70.37%, and increasing the scaffold N50 by 49.73%.

**Figure 2.**
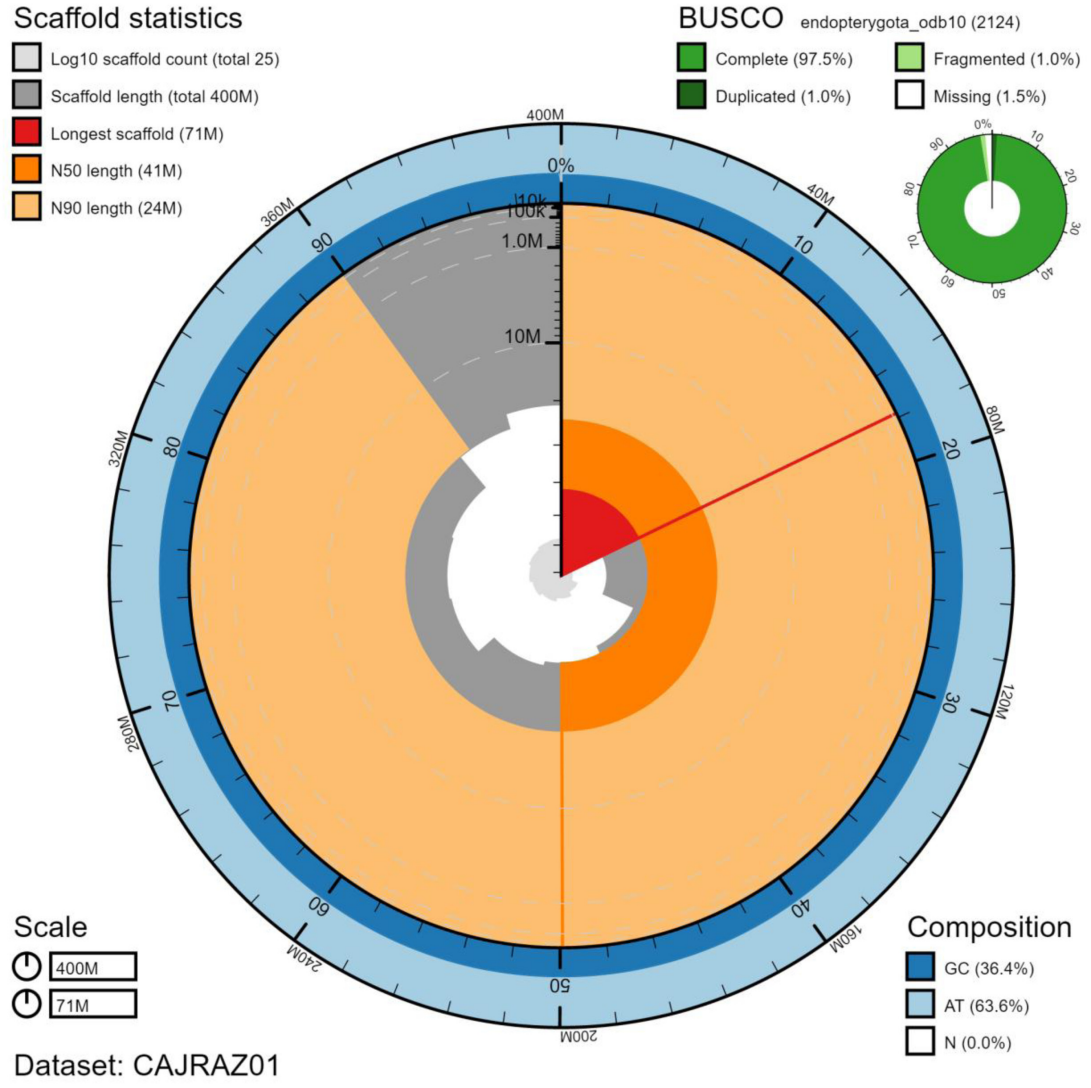
Genome assembly of *Coccinella septempunctata*, icCocSept1.1: metrics. The main plot is divided into 1,000 size-ordered bins around the circumference with each bin representing 0.1% of the 398,868,586 bp assembly. The distribution of scaffold lengths is shown in dark grey with the plot radius scaled to the longest scaffold present in the assembly (71,177,040 bp, shown in red). Orange and pale-orange arcs show the N50 and N90 scaffold lengths (41,442,133 and 24,000,787 bp), respectively. The pale grey spiral shows the cumulative scaffold count on a log scale with white scale lines showing successive orders of magnitude. The blue and pale-blue area around the outside of the plot shows the distribution of GC, AT and N percentages in the same bins as the inner plot. A summary of complete, fragmented, duplicated and missing BUSCO genes in the endopterygota_odb10 set is shown in the top right. An interactive version of this figure is available at https://blobtoolkit.genomehubs.org/view/icCocSept1.1/dataset/CAJRAZ01/snail.

The final assembly has a total length of 399 Mb in 24 sequence scaffolds with a scaffold N50 of 41.4 Mb ( Table 1). The majority, 99.96%, of the assembly sequence was assigned to 10 chromosomal-level scaffolds, representing 9 autosomes (numbered by sequence length), and the X sex chromosome ( Figure 2– Figure 5; Table 2). There is a repeat that is shared between chromosomes X and 9 that could be slightly differently distributed between the two. The assembly has a BUSCO v5.1.2 ( Manni *et al.*, 2021) completeness of 97.5% (single 96.4%, duplicated 1.0%) using the endopterygota_odb10 reference set. While not fully phased, the assembly deposited is of one haplotype. Contigs corresponding to the second haplotype have also been deposited.

**Table 1.** Genome data for *Coccinella septempunctata*, icCocSept1.1.

*Project accession data*	
Assembly identifier	icCocSept1.1
Species	*Coccinella septempunctata*
Specimen	icCocSept1
NCBI taxonomy ID	NCBI:txid41139
BioProject	PRJEB44834
BioSample ID	SAMEA7520205
Isolate information	Female, whole organism
*Raw data accessions*	
PacificBiosciences SEQUEL II	ERR6436372, ERR6558185
10X Genomics Illumina	ERR6054712-ERR6054715
Hi-C Illumina	ERR6054716
*Genome assembly*	
Assembly accession	GCA_907165205.1
Accession of alternate haplotype	GCA_907165185.1
Span (Mb)	399
Number of contigs	110
Contig N50 length (Mb)	16.5
Number of scaffolds	24
Scaffold N50 length (Mb)	41.4
Longest scaffold (Mb)	71.2
BUSCO * genome score	C:97.5%[S:96.4%,D:1.0%],F:1.0%,M:1.5%,n:2124

*BUSCO scores based on the endopterygota_odb10 BUSCO set using v5.1.2. C= complete [S= single copy, D=duplicated], F=fragmented, M=missing, n=number of orthologues in comparison. A full set of BUSCO scores is available at https://blobtoolkit.genomehubs.org/view/icCocSept1.1/dataset/CAJRAZ01/busco.

**Figure 3.**
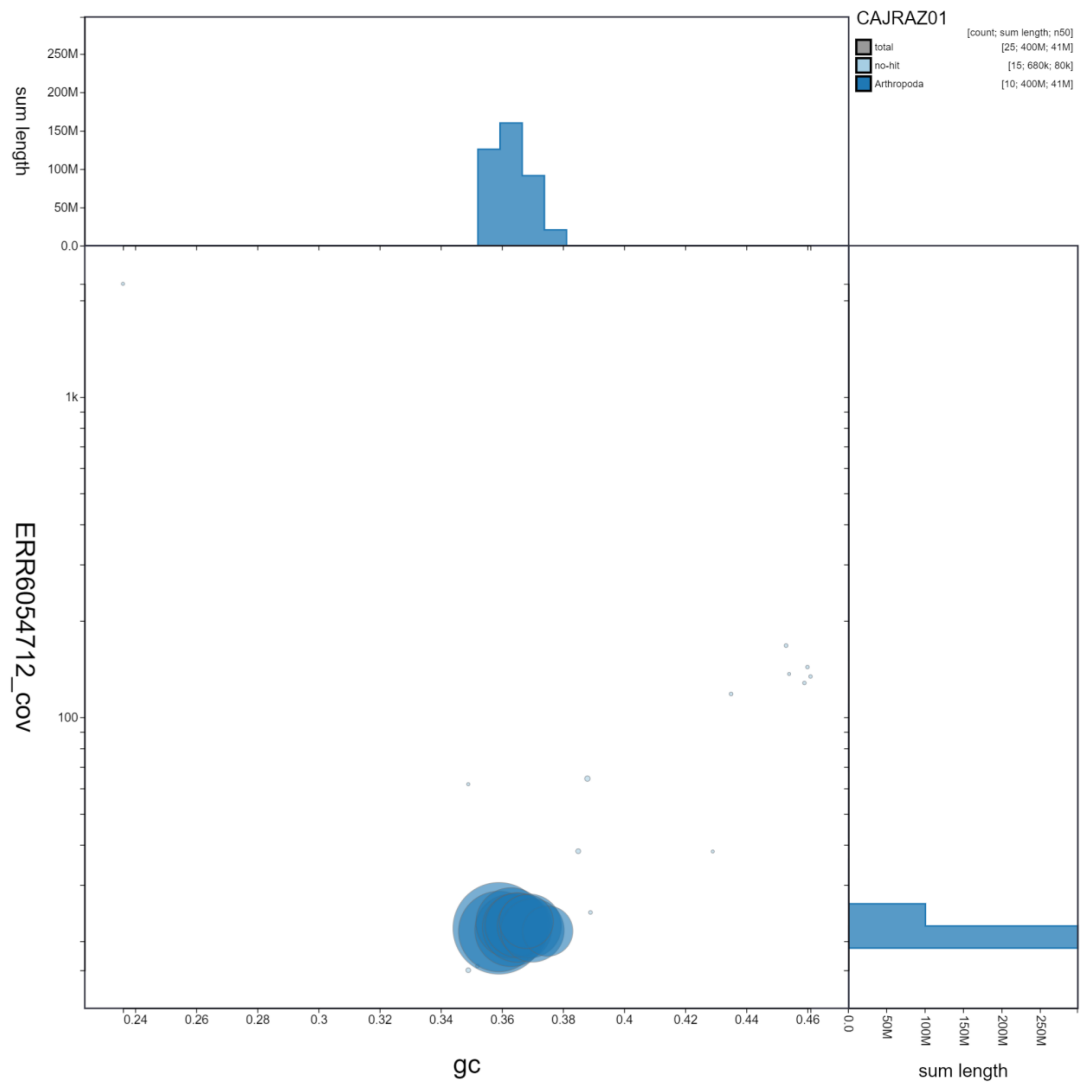
Genome assembly of *Coccinella septempunctata*, icCocSept1.1: GC coverage. BlobToolKit GC-coverage plot. Scaffolds are coloured by phylum. Circles are sized in proportion to scaffold length Histograms show the distribution of scaffold length sum along each axis. An interactive version of this figure is available at https://blobtoolkit.genomehubs.org/view/icCocSept1.1/dataset/CAJRAZ01/blob.

**Figure 4.**
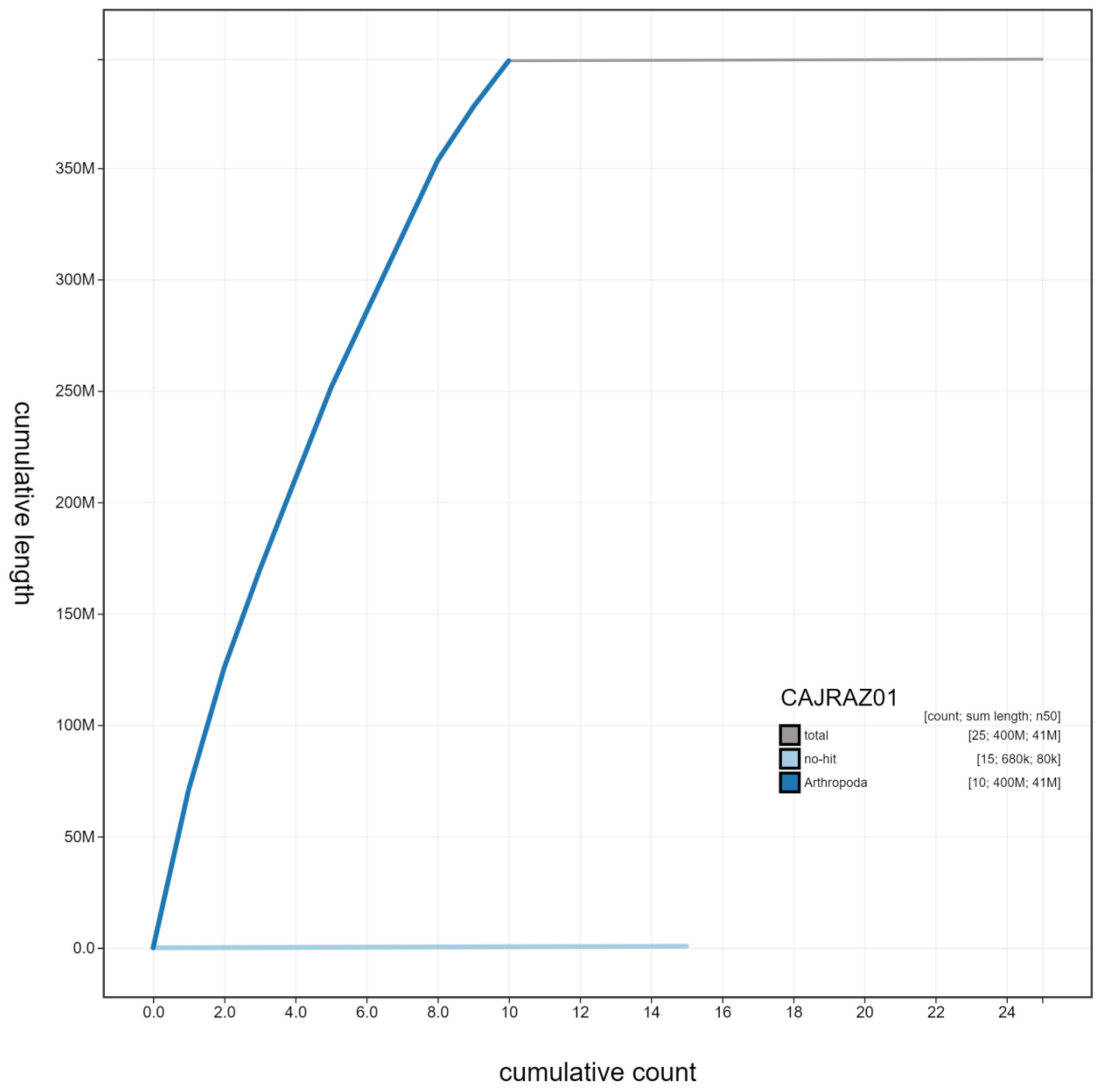
Genome assembly of *Coccinella septempunctata*, icCocSept1.1: cumulative sequence. BlobToolKit cumulative sequence plot. The grey line shows cumulative length for all scaffolds. Coloured lines show cumulative lengths of scaffolds assigned to each phylum using the buscogenes taxrule. An interactive version of this figure is available at https://blobtoolkit.genomehubs.org/view/icCocSept1.1/dataset/CAJRAZ01/cumulative.

**Figure 5.**
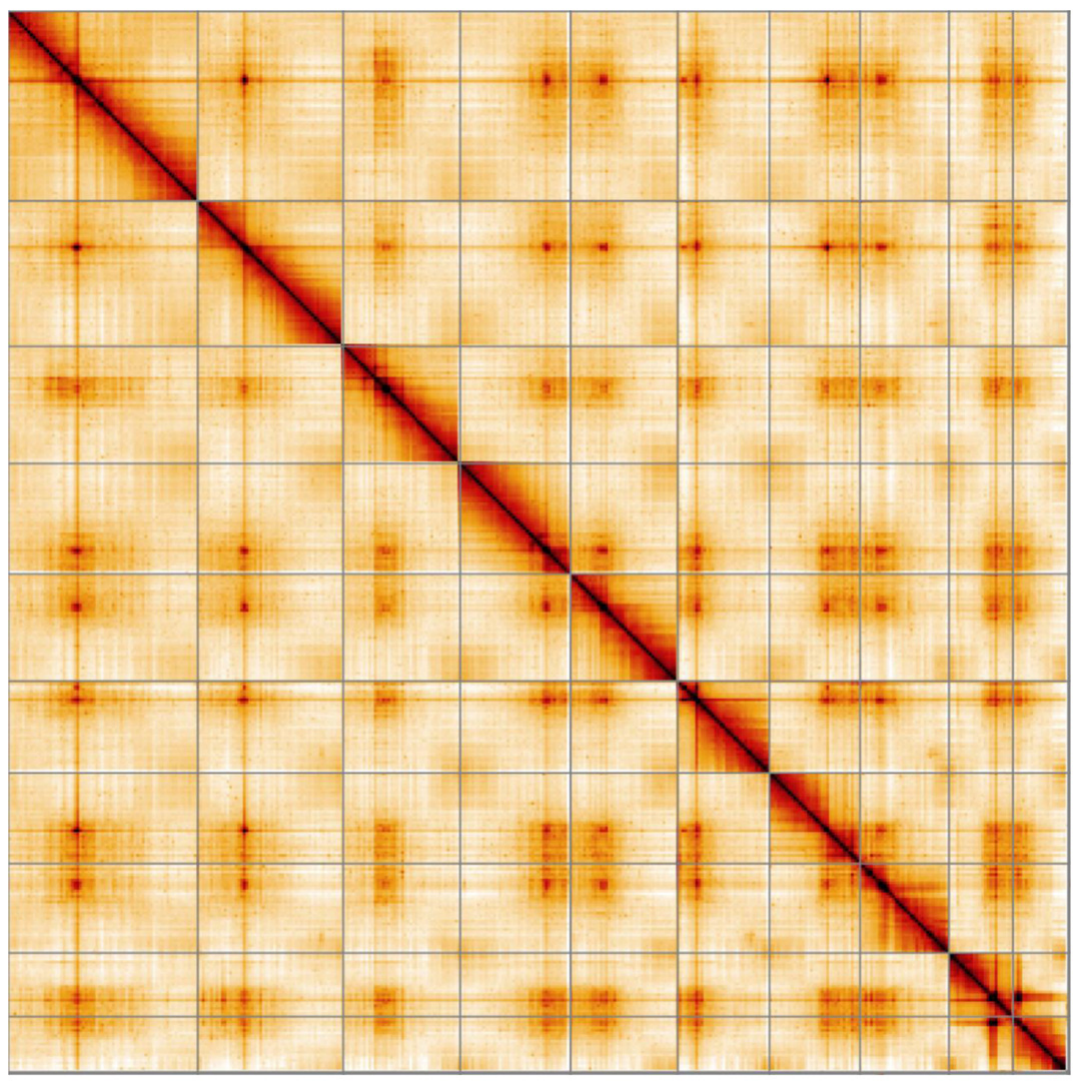
Genome assembly of *Coccinella septempunctata*, icCocSept1.1: Hi-C contact map. Hi-C contact map of the icCocSept1.1 assembly, visualised in HiGlass.

**Table 2.** Chromosomal pseudomolecules in the genome assembly of *Coccinella septempunctata*, icCocSept1.1.

INSDC accession	Chromosome	Size (Mb)	GC%
OU015573.1	1	71.18	35.9
OU015574.1	2	54.65	35.9
OU015575.1	3	43.94	36.3
OU015576.1	4	41.44	36.3
OU015577.1	5	40.26	36.5
OU015578.1	6	34.46	36.5
OU015579.1	7	34.01	36.9
OU015580.1	8	33.49	37
OU015582.1	9	20.75	37.5
OU015581.1	X	24.00	36.8
OU015583.1	MT	0.02	23.7
-	Unplaced	0.66	39.7

## Methods

### Sample acquisition and nucleic acid extraction

A single female *C. septempunctata* was collected from Wytham Farm, Oxfordshire (biological vice-county: Berkshire), UK (latitude 51.779, longitude -0.317) by Liam Crowley, University of Oxford, using a pooter. The sample was identified by the same individual and snap-frozen on dry ice.

DNA was extracted from the whole organism of icOcyOlen1 at the Wellcome Sanger Institute (WSI) Scientific Operations core from the whole organism using the Qiagen MagAttract HMW DNA kit, according to the manufacturer’s instructions. Following this, further DNA was extracted for a PacBio top-up. Tissue was cryogenically disrupted to a fine powder using a Covaris cryoPREP Automated Dry Pulveriser, receiving multiple impacts. Fragment size analysis of 0.01-0.5 ng of DNA was then performed using an Agilent FemtoPulse. High molecular weight (HMW) DNA was again extracted using the Qiagen MagAttract HMW DNA extraction kit. HMW DNA was sheared into an average fragment size between 12–20 kb in a Megaruptor 3 system with speed setting 30. Sheared DNA was purified by solid-phase reversible immobilisation using AMPure PB beads with a 1.8X ratio of beads to sample to remove the shorter fragments and concentrate the DNA sample. The concentration of the sheared and purified DNA was assessed using a Nanodrop spectrophotometer and Qubit Fluorometer and Qubit dsDNA High Sensitivity Assay kit. Fragment size distribution was evaluated by running the sample on the FemtoPulse system.

### Sequencing

Pacific Biosciences HiFi circular consensus and 10X Genomics read cloud DNA sequencing libraries were constructed according to the manufacturers’ instructions. Sequencing was performed by the Scientific Operations core at the Wellcome Sanger Institute on Pacific Biosciences SEQUEL II and Illumina HiSeq X instruments. Hi-C data were generated using the Arima v2 Hi-C kit and sequenced on an Illumina NovaSeq 6000 instrument.

### Genome assembly

Assembly was carried out with HiCanu ( Nurk *et al.*, 2020); haplotypic duplication was identified and removed with purge_dups ( Guan *et al.*, 2020). One round of polishing was performed by aligning 10X Genomics read data to the assembly with longranger align, calling variants with freebayes ( Garrison & Marth, 2012). The assembly was then scaffolded with Hi-C data ( Rao *et al.*, 2014) using SALSA2 ( Ghurye *et al.*, 2019). The assembly was checked for contamination and corrected using the gEVAL system ( Chow *et al.*, 2016) as described previously ( Howe *et al.*, 2021). Manual curation ( Howe *et al.*, 2021) was performed using gEVAL, HiGlass ( Kerpedjiev *et al.*, 2018) and Pretext. The mitochondrial genome was assembled using MitoHiFi ( Uliano-Silva *et al.*, 2021). The genome was analysed and BUSCO scores generated within the BlobToolKit environment ( Challis *et al.*, 2020). Table 3 contains a list of all software tool versions used, where appropriate.

**Table 3.** Software tools used.

Software tool	Version	Source
HiCanu	2.1	Nurk *et al.*, 2020
purge_dups	1.2.3	Guan *et al.*, 2020
SALSA2	2.2	Ghurye *et al.*, 2019
longranger align	2.2.2	https://support.10xgenomics.com/genome-exome/software/pipelines/latest/advanced/other-pipelines
freebayes	1.3.1-17-gaa2ace8	Garrison & Marth, 2012
MitoHiFi	1.0	Uliano-Silva *et al.*, 2021
gEVAL	N/A	Chow *et al.*, 2016
HiGlass	1.11.6	Kerpedjiev *et al.*, 2018
PretextView	0.1.x	https://github.com/wtsi-hpag/PretextView
BlobToolKit	2.6.2	Challis *et al.*, 2020

### Ethics/compliance issues

The materials that have contributed to this genome note have been supplied by a Darwin Tree of Life Partner. The submission of materials by a Darwin Tree of Life Partner is subject to the Darwin Tree of Life Project Sampling Code of Practice. By agreeing with and signing up to the Sampling Code of Practice, the Darwin Tree of Life Partner agrees they will meet the legal and ethical requirements and standards set out within this document in respect of all samples acquired for, and supplied to, the Darwin Tree of Life Project. Each transfer of samples is further undertaken according to a Research Collaboration Agreement or Material Transfer Agreement entered into by the Darwin Tree of Life Partner, Genome Research Limited (operating as the Wellcome Sanger Institute), and in some circumstances other Darwin Tree of Life collaborators.

## Data availability

European Nucleotide Archive: Coccinella septempunctata (seven-spotted ladybird). Accession number PRJEB44834; https://identifiers.org/ena.embl/PRJEB44834

The genome sequence is released openly for reuse. The *C. septempunctata* genome sequencing initiative is part of the Darwin Tree of Life (DToL) project. All raw sequence data and the assembly have been deposited in INSDC databases.The genome will be annotated and presented through the Ensembl pipeline at the European Bioinformatics Institute. Raw data and assembly accession identifiers are reported in Table 1.

## Author information

Members of the University of Oxford and Wytham Woods Genome Acquisition Lab are listed here: https://doi.org/10.5281/zenodo.4789929.

Members of the Darwin Tree of Life Barcoding collective are listed here: https://doi.org/10.5281/zenodo.4893704.

Members of the Wellcome Sanger Institute Tree of Life programme collective are listed here: https://doi.org/10.5281/zenodo.5377053.

Members of Wellcome Sanger Institute Scientific Operations: DNA Pipelines collective are listed here: https://doi.org/10.5281/zenodo.4790456.

Members of the Tree of Life Core Informatics collective are listed here: https://doi.org/10.5281/zenodo.5013542.

Members of the Darwin Tree of Life Consortium are listed here: https://doi.org/10.5281/zenodo.4783559.
