# Supplementary material for: The genome sequence of the seven-spotted ladybird, Coccinella septempunctata Linnaeus, 1758
Source: Wellcome Open Res. 2021 Nov 24;6:319. [Version 1] doi: 10.12688/wellcomeopenres.17346.1 (PMC8630551; doi:10.12688/wellcomeopenres.17346.1)
Supplement: Peer review history

# Peer review: Reviewer response for version 1

**DOI:** 10.21956/wellcomeopenres.19176.r47194

**Published:** 2021-11-29

**Authors:** Jongsun Park

**Affiliations:** 1 InfoBoss Inc, Seoul, South Korea

The manuscript entitled "The genome sequence of the seven-spotted ladybird, *Coccinella septempunctata *Linnaeus, 1758" describe the whole genome sequence of *C. septempunctata*. All genomic data are properly available in the database and the figures which describe features of the whole genome assembly are properly drawn.

 I'm not sure whether the author can also provide gene model of this genome or not because NCBI provides a number of transcripts. I think that just brief information of the gene model of this genome wiould be better for this manuscript.

Are sufficient details of methods and materials provided to allow replication by others?

Yes

Is the rationale for creating the dataset(s) clearly described?

Yes

Are the datasets clearly presented in a useable and accessible format?

Yes

Are the protocols appropriate and is the work technically sound?

Yes

Reviewer Expertise:

Genomics, Comparative Genomics, Bioinformatics, Biodiversity informatics, Plant Taxonomy

I confirm that I have read this submission and believe that I have an appropriate level of expertise to confirm that it is of an acceptable scientific standard.

# Peer review: Reviewer response for version 1

**DOI:** 10.21956/wellcomeopenres.19176.r47192

**Published:** 2021-11-25

**Authors:** Stephen Richards

**Affiliations:** 1 Human Genome Sequencing Center, Department of Human and Molecular Genetics, Baylor College of Medicine, Houston, TX, USA

The is great genome assembly, of an important species, and I can easily find the data at NCBI and EBI - all of this is good.

 My only tiny issue (and it is a tiny tiny issue) is that although the methods are described in the standard manner, and the software versions are used, there could be a better way - perhaps with a document showing example commands and a continuer to download the software - perhaps this needs to be a methods publication for this set of papers that really help a graduate student repeat the assembly?

 Otherwise, a wonderful high quality assembly that has made entomology a better place.

Are sufficient details of methods and materials provided to allow replication by others?

Partly

Is the rationale for creating the dataset(s) clearly described?

Yes

Are the datasets clearly presented in a useable and accessible format?

Yes

Are the protocols appropriate and is the work technically sound?

Yes

Reviewer Expertise:

Arthropod Genomics, Human Genomics, transcriptomics

I confirm that I have read this submission and believe that I have an appropriate level of expertise to confirm that it is of an acceptable scientific standard.
